# A single nucleotide polymorphism genotyping platform for the authentication of patient derived xenografts

**DOI:** 10.18632/oncotarget.11125

**Published:** 2016-08-09

**Authors:** Jad El-Hoss, Duohui Jing, Kathryn Evans, Cara Toscan, Jinhan Xie, Hyunjoo Lee, Renea A. Taylor, Mitchell G. Lawrence, Gail P. Risbridger, Karen L. MacKenzie, Rosemary Sutton, Richard B. Lock

**Affiliations:** ^1^ Children's Cancer Institute, Lowy Cancer Research Centre, Sydney, UNSW, Australia; ^2^ Prostate Research Group, Department of Physiology, Biomedicine Discovery Institute, Monash Partners Comprehensive Cancer Consortium, Monash University, Clayton, VIC, Australia; ^3^ Prostate Research Group, Department of Anatomy and Developmental Biology, Biomedicine Discovery Institute, Monash Partners Comprehensive Cancer Consortium, Monash University, Clayton, VIC, Australia

**Keywords:** SNP genotyping, patient derived xenografts, authentication, OpenArray, R studio

## Abstract

Patient derived xenografts (PDXs) have become a vital, frequently used, component of anti-cancer drug development. PDXs can be serially passaged *in vivo* for years, and shared across laboratories. As a consequence, the potential for mis-identification and cross-contamination is possible, yet authentication of PDXs appears limited. We present a PDX Authentication System (PAS), by combining a commercially available OpenArray assay of single nucleotide polymorphisms (SNPs) with in-house R studio programs, to validate PDXs established in individual mice from acute lymphoblastic leukemia biopsies. The PAS is sufficiently robust to identify contamination at levels as low as 3%, similar to the gold standard of short tandem repeat (STR) profiling. We have surveyed a panel of PDXs established from 73 individual leukemia patients, and found that the PAS provided sufficient discriminatory power to identify each xenograft. The identified SNP-discrepant PDXs demonstrated distinct gene expression profiles, indicating a risk of contamination for PDXs at high passage number. The PAS also allows for the authentication of tumor cells with complex karyotypes from solid tumors including prostate cancer and Ewing's sarcoma. This study highlights the demands of authenticating PDXs for cancer research, and evaluates a reliable authentication platform that utilizes a commercially available and cost-effective system.

## INTRODUCTION

Patient derived xenografts (PDXs) are a vital tool for the pre-clinical evaluation of new drugs [1–6]. PDXs are established from patient biopsies by engraftment into immunodeficient mice, thereby allowing for pre-clinical animal testing [7–12]. The validation of PDX models has been the focus of extensive research, primarily to confirm that they faithfully reproduce the primary disease state [13]. In contrast, the need to authenticate PDXs (whereby PDXs are confirmed to be pure and derived from the original patient biopsy) has been largely overlooked, resulting in potentially misleading reports and mis-management of patient samples. With the increasing use of PDXs in cancer research, implementation of a rapid, accurate and cost-effective genotyping system for PDX authentication would improve the integrity of data produced from PDXs.

Short tandem repeats (STRs) are repetitive sequences of DNA that are distributed throughout the human genome [14–16]. STRs have a high mutation rate that results in a large diversity of repetitive sequences across the human population. The ability to quantify repetitive elements and their diversity in the population has resulted in the implementation of STRs for the purpose of human identification across forensic science, cell line authentication, and in limited instances authentication of PDXs [17]. The high demand for this technology and its widespread use has resulted in commercially available kits for profiling and authentication, and is now a requirement for publication of cell lines in many journals.

The analysis of single nucleotide polymorphisms (SNPs) within cell populations has become increasingly attractive as a method for cell line authentication [18–21]. SNPs offer a similar level of accuracy to STRs for correctly identifying cell lines. Their much lower mutation rate and higher throughput with automated analysis led to proposals to use SNPs for the authentication of cell lines [22–24]. Even though cell lines are often profiled by STR, PDXs generally have not been routinely profiled to date, which provides an opportunity for SNP-based authentication. PDXs are often the final step prior to clinical evaluation of a novel drug, so it is important to authenticate these samples in a rapid, accurate and cost-effective manner.

Herein we describe the implementation of a SNP-based PDX-Authentication-System (PAS), that utilizes a commercially available, high-throughput, and cost-effective SNP genotyping platform and in-house R studio programs, to test a large panel of PDXs derived from pediatric acute lymphoblastic leukemia (ALL) and solid tumor (e.g. prostate cancer and Ewing's sarcoma) patient samples. The detection threshold for this method was found to be comparable with STR profiling. In particular, we found that patient-derived samples exhibiting complex karyotypes (commonly found in cancer cells [25–27]) or even chimerism, could be identified using this technology.

## RESULTS

### Potential causes of PDX mis-identification

**Figure 1:**
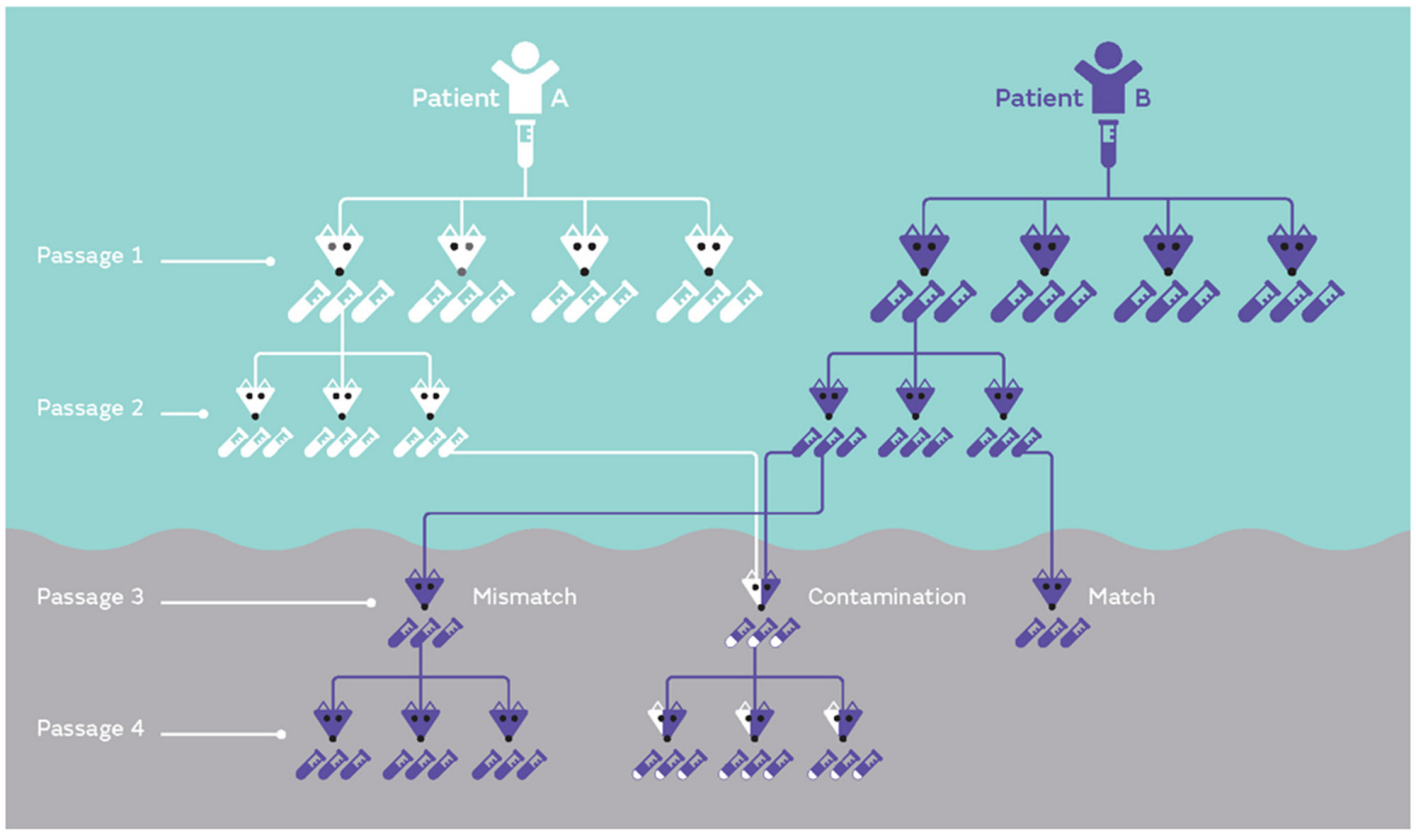
Examples of PDX mis-identification PDXs established from either patient A or B are inoculated into a suitable number of mice (four mice in this example at Passage 1). Once the mice reach a defined endpoint, each mouse contributes a large number of samples to expand the biobank (each mouse contributes three samples in this example). Each sample can be serially expanded, as shown in Passage 2. Potential common mistakes involve either mis-labeling (or mismatch) of samples or contamination of one sample with another sample (shown at Passage 3). If mis-identification or contamination is not corrected early, samples can be serially expanded (Passage 4). In the case of contamination if this is not identified early on in the process, a mix of equal parts can lead to competition and a dominant sample can take over (Passage 4).

Although there are a number of scenarios that could potentially lead to PDX mis-identification, two common errors are illustrated in Figure 1. These include the mis-identification of a sample (due to labeling or communication errors), or the cross-contamination of two or more PDXs (possibly due to carry over of samples during harvest). Both examples are illustrated at Passage 3. If a sample derived from Patient B is mis-identified as belonging to Patient A, this can be carried over in subsequent expansions and experiments (Passage 4). Alternatively, if a sample is contaminated with two PDXs (for example an equal mix of Patient A and Patient B at Passage 3), both samples will compete in the mouse and one sample may eventually dominate the other (for example Patient B dominated Patient A in Passage 4). In contrast, a pure and matched PDX sample is also illustrated at Passage 3, which maintains characteristics of leukemia cells from Patient B.

### Reference profiles for PDXs

**Figure 2:**
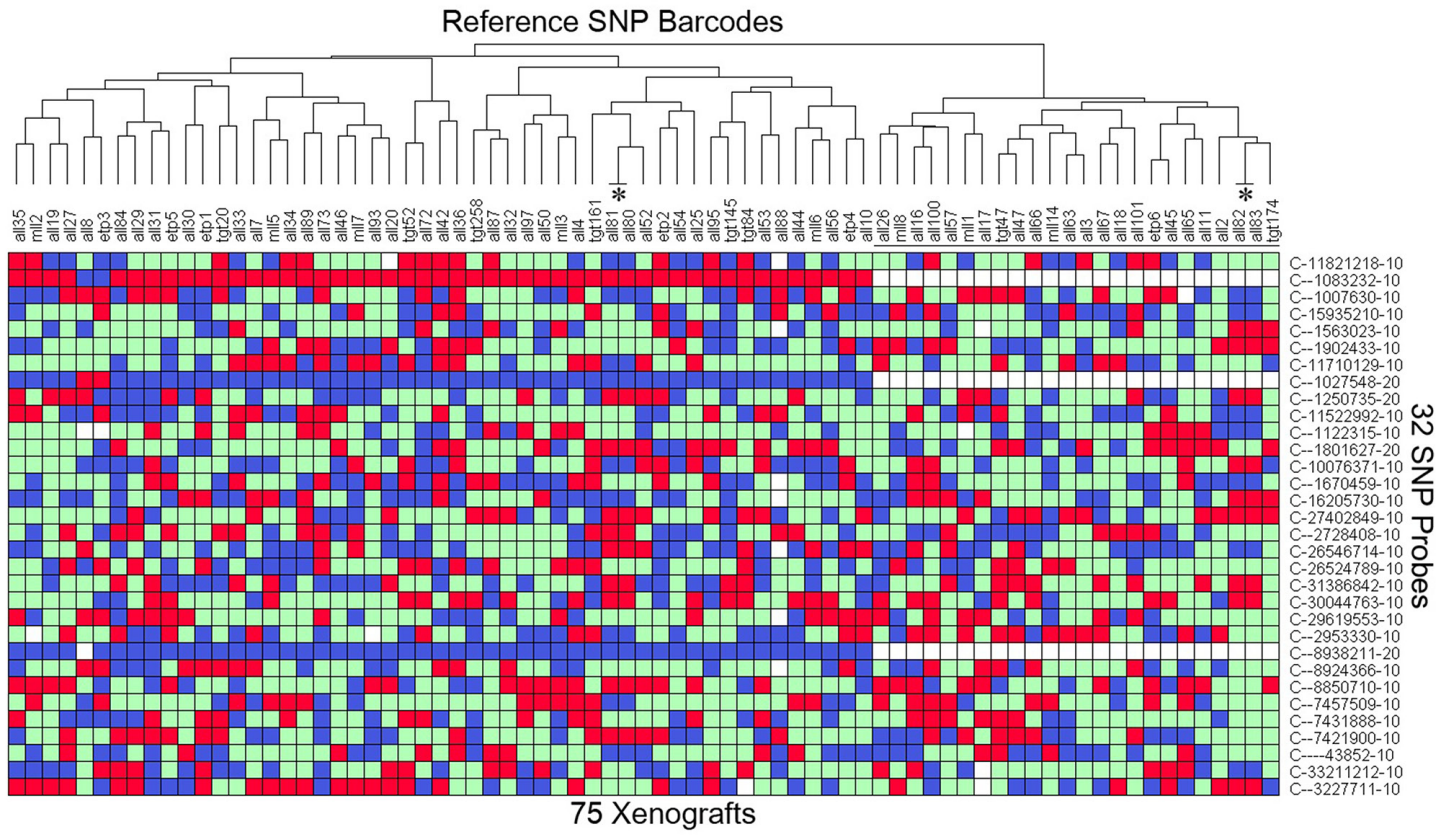
Heatmap of the SNP profile of 75 established PDXs DNA was extracted from the earliest available sample from 73 patients (typically the patient sample), and SNP genotyped on the Taqman OpenArray 32A Barcode. This provides the reference profile for all the PDXs. All 32 SNPs are bi-allelic resulting in a Homozygous Allele 1 (red), Homozygous Allele 2 (blue), Heterozygous (green), or no amplification (white) reading. Samples are clustered according to the SNP TaqMan assay (Y-axis) to identify any matches. The 24 female samples are labeled with a black line. 2 pairs of PDXs (labeled with *, ALL-80/-81 and ALL-82/-83) match as expected because they are derived from the same patient. Any PDX can be cross-referenced to this table to identify a mismatch. The details of the SNP genotype for all PDXs are provided in Supplementary Table S1.

To develop a panel of reference barcodes for each patient/PDX, genomic DNA was harvested from patient samples used for the establishment of the PDX, or if unavailable, a sample from the earliest available PDX passage. Samples were genotyped at the 32 SNPs of interest, and a barcode was generated for each sample (Figure 2). Hierarchical clustering confirmed that no two PDXs shared the same profile. Two pairs of these PDXs (as labeled with *) were derived from paired samples of the same patient (i.e. diagnosis and relapse sample), and each pair showed an identical match. Three SNPs locating at the Y chromosome, i.e. C___1027548_10, C___8938211_10 and C___1083232_10, were absent in all female samples (Figure 2). All cells harvested from subsequently xenografted mice were validated to ensure they matched the expected SNP reference profile. The detailed SNP profile for all PDXs is available in Supplemental Table S1.

### SNP genotyping in contamination identification

**Figure 3:**
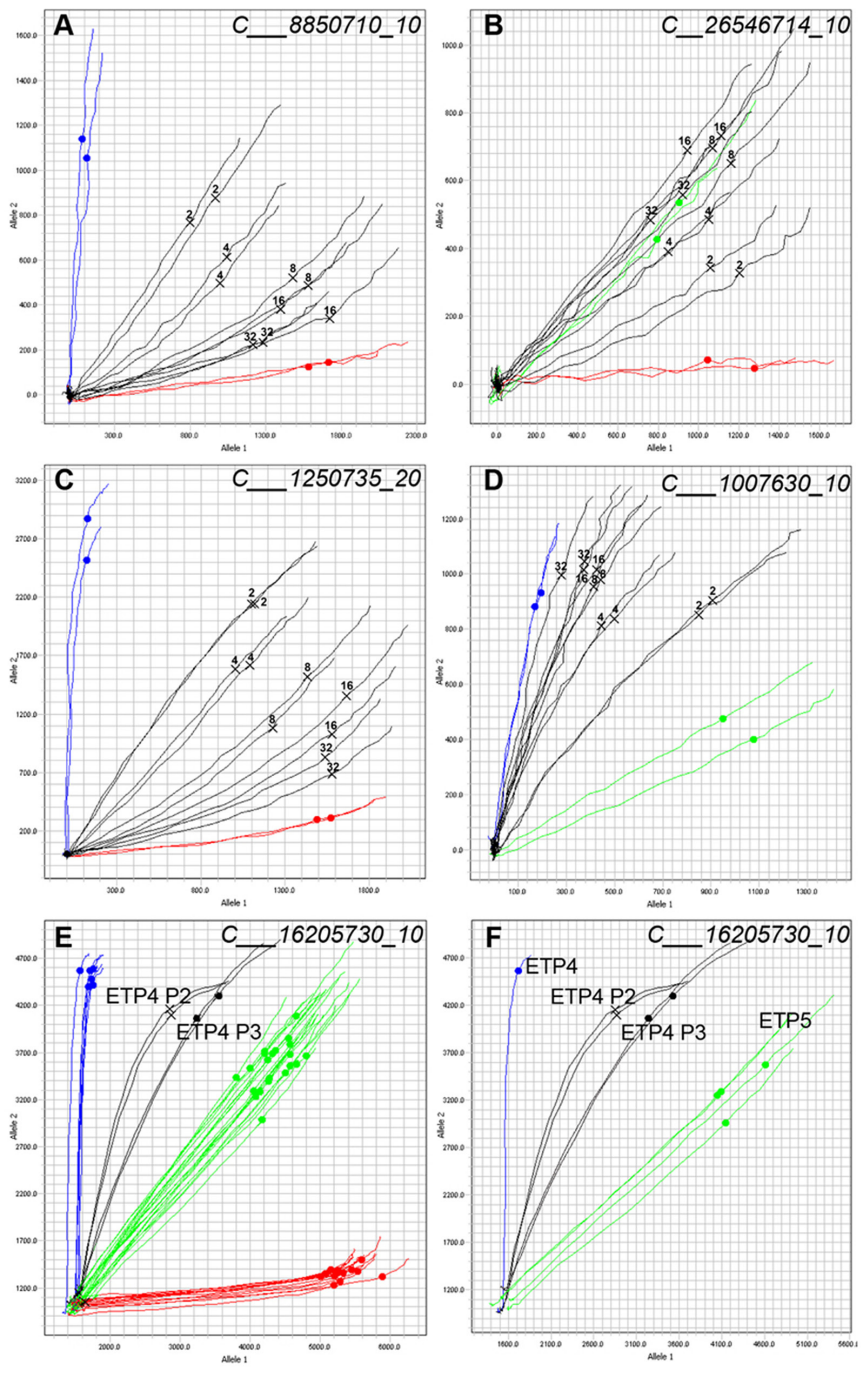
Allelic discrimination plot analysis allows for the detection of contaminated samples Samples of ALL-10 and ALL-19 (A and B) or ALL-46 and ALL-35 (C and D) were mixed at ratios of 1:1 (2), 1:3 (4), 1:7 (8), 1:15 (16), and 1:31 (32) and SNP genotyped. Two representative SNPs are shown. Homozygous Allele 1 amplifies on the X axis (red), Homozygous Allele 2 amplifies on the Y axis (blue), and Heterozygous samples amplify such that X = Y (green). Each line represents the real-time amplification analysis for an individual sample. All samples are run in duplicate. **A.** Mixed ratios of ALL-10 (blue) and ALL-19 (red) with the known mixtures labeled and shown in black. ALL-19 is the major component. **B.** Mixed ratios of ALL-10 (red) and ALL-19 (green) with the known mixtures labeled in black. **C.** Mixed ratios of ALL-46 (blue) and ALL-35 (red) with the known mixtures in black. **D.** Mixed ratios of ALL-46 (green) and ALL-35 (blue) with known mixtures shown in black. (**E** and **F.**) Allelic discrimination plots of a contaminated PDX sample. Sample from Passage 2 of PDX ETP-4 (ETP-4 P2, black, two samples run in duplicate) shows signs of mixture, and the same sample serially expanded into another mouse yielded the profile in ETP-4 P3 (black) at Passage 3. **E.** includes a panel of PDXs from several different patients; **F.** shows ETP-4 (blue) and ETP-5 (green) only.

The allelic discrimination plots of individual SNPs allows for the identification of samples that are a mixture of two or more PDXs. The evaluation of mixed samples was performed by mixing DNA of two PDXs at fixed ratios. PDXs ALL-10 and ALL-19 (Figure 3A and 3B) or ALL-46 and ALL-35 (Figure 3C and 3D) were serially mixed in ratios of 1:1, 1:3, 1:7, 1:15 and 1:31.

In cases where the two pure samples are homozygous for the opposite allele (Figure 3A and 3C), the 1:1 mix appears as a heterozygous SNP. Detection of a sample contaminated at 1:31 can be identified as the multicomponent curve shifts away from the pure homozygous sample, suggesting the late amplification of the opposite allele. Yet, at SNP loci with a heterozygote allele in one sample (Figure 3B and 3D) the identification of a mixture is more challenging, and the detection of contamination is limited in highly diluted mixtures. When identifying a mixture in a sample, it is the integrated analysis of the allelic discrimination plots for all 32 SNPs that is revealing, and in both cases illustrated, the mixture could be easily identified at a ratio of 1:31.

Contamination of a sample can lead to competition between two PDXs, and the potential disappearance of the original PDX. A contaminated PDX ETP-4 at Passage 2 (ETP-4 P2) was expanded into Passage 3 (ETP-4 P3) and the shift is visible in the allelic discrimination plot between the two passages (Figure 3E and 3F). Further SNP and STR analysis (data not shown) confirmed that the contaminant was PDX ETP-5. In this example, it is clear that ETP-4 P3 has shifted towards ETP-5, and that the contaminant is out-competing the original PDX. Identification of contaminants allows for problematic samples and data to be discarded appropriately.

### Using PAS to identify contaminated PDXs

We have established a platform, i.e. PDX-Authentication-System (PAS), by combining a commercially available TaqMan 32A OpenArray platform with our in-house R studio programs, to validate PDXs derived from every individual mouse used in our group. In this study, we performed a representative analysis on a panel of 1 patient sample and 74 PDXs derived from the same patient ALL-19, including: 6 xenografts of passage 3, 6 xenografts of passage 4, and 62 xenografts of passage 5, as described in Table 1 and Supplemental Table S2.

**Table 1:** Summary of ALL-19 PDXs determined in SNP genotyping. Each sample represents an ALL-19 xenograft derived from an individual mouse

	Sample numbers	Executive ID	Contaminated sample counts
**Passage 1**	1 (patient sample)	P1	0
**Passage 3**	6	P3-1 to P3-6	0
**Passage 4**	6	P4-1 to P4-6	0
**Passage 5**	62	P5-1 to P5-62	8 (P5-15, P5-16, P5-18, P5-21, P5-22, P5-24, P5-25, P5-26)

**Figure 4:**
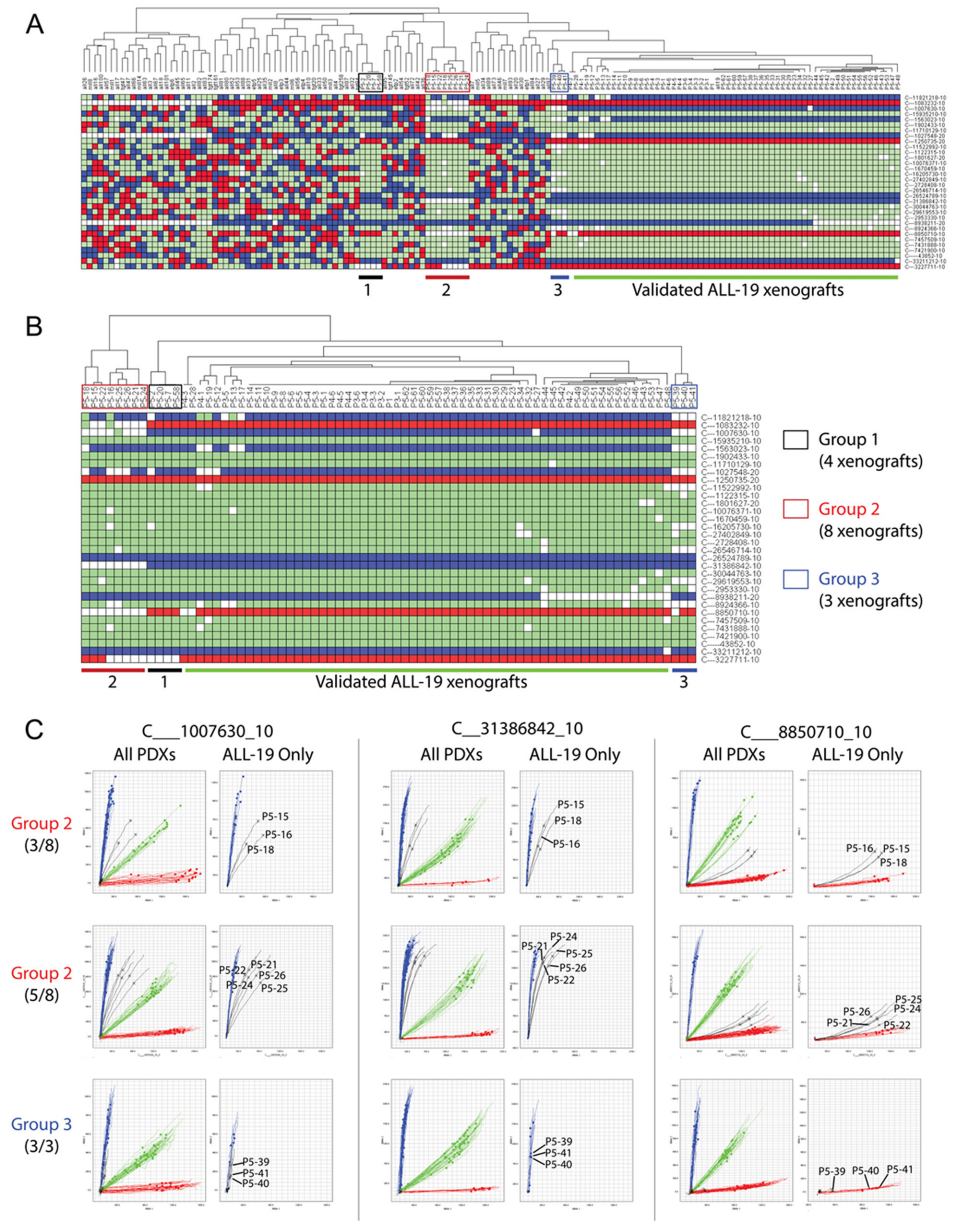
Identification of contaminated PDXs from a panel of ALL-19 PDXs derived from 74 individual mice **A.** Clustering of the 74 ALL-19 PDXs and 1 patient sample with the Reference SNP profiles. The ALL-19 PDXs are separated into 4 groups, including Groups 1, 2, 3 and a validated group. In the heatmap, red refers to Homozygous Allele 1, blue refers to Homozygous Allele 2, green refers to Heterozygous, and white refers to no amplification or undetermined PCR. **B.** Hierarchical clustering of only the ALL-19 PDXs based on SNP genotypes. **C.** Allelic discrimination plots of 3 representative SNP probes in Group 2 (8 PDXs at passage 5) and Group 3 (3 PDXs at passage 5). The details of the SNP profiles of the patient sample and ALL-19 PDXs engrafted in 74 mice are provided in Supplementary Table S2.

Barcoding these samples with the 32 SNP probes generated SNP profiles of ALL-19 PDX engrafted in 74 NOD/SCID mice and one patient sample (Supplemental Table S2). These were clustered with the reference SNP profiles from Figure 2. Sixty xenografts, including all xenografts from low passages (i.e. P1, P3 and P4), are clustered together and match with the reference ALL-19 profile - these are considered to be validated (Figure 4A). However, 3 groups from passage 5 reveal discrepant profiles and locate away from the validated ALL-19 group in Hierarchical Clustering. Focusing on the ALL-19 PDXs, we found that the 3 groups maintained separation from the validated group and were significantly enriched with undetermined genotypes (white squares; Figure 4B).

Within PAS, we proposed to further verify the undetermined genotypes in the potentially contaminated groups with integrated analyses of allelic discrimination plots for all 32 SNPs. Plots of three representative SNP probes in PDXs from Group 2 and 3 are demonstrated in Figure 4C. Amplification curves from the 8 PDXs in Group 2 shift away from the pure homozygous allele amplifications (Allele 2 for C___1007630_10 and C__31386842_10; Allele 1 for C___8850710_10), indicating contamination in these samples. In addition, all the 8 PDXs were recorded to be generated from the same experiment. However, the amplification curves from the 3 PDXs in Group 3 still match with the pure homozygous allele amplification, even though amplification of some samples (all 3 samples with C___1007630_10 and P5-39 with C___8850710_10) are not as robust as that of other PDXs. This indicates that the undetermined genotypes of the Group 3 PDXs are likely due to inefficient amplification rather than contamination. For a similar reason, the Group 1 PDXs reveal no contamination either (data not shown). Overall, 8 passage-5 ALL-19 PDXs (Group 2) indicate contamination, over the course of a decade of routine lab work.

### Characterizing SNP-discrepant PDXs

**Figure 5:**
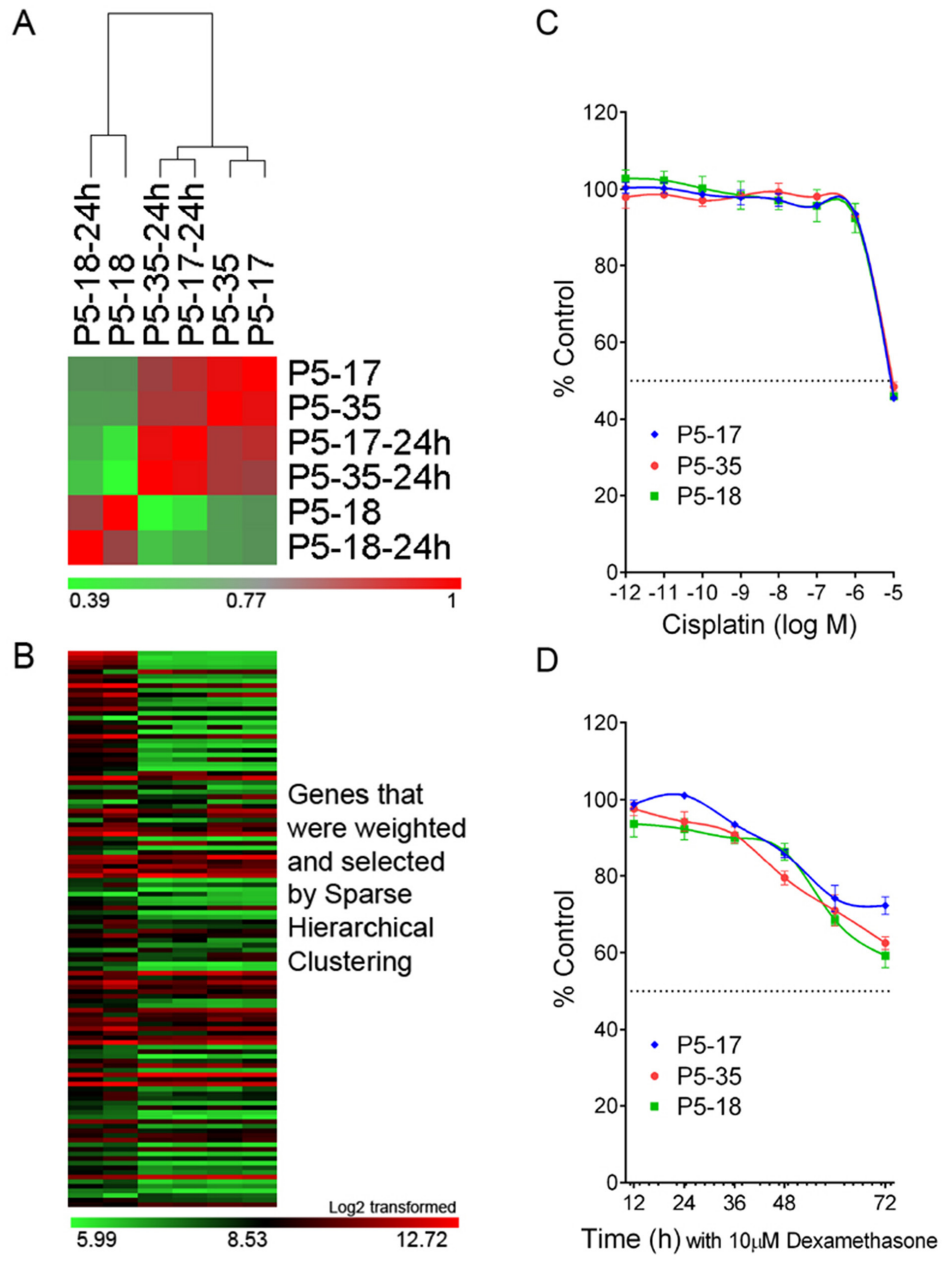
Gene expression analysis and cytotoxicity assays on the SNP-discrepant ALL-19 PDXs **A.** Correlation analysis on ALL-19 PDXs, including 2 validated (P5-17 and P5-35) and 1 contaminated (P5-18) sample. “-24h” refers to PDXs incubated in QBSF 60/F *in vitro* for 24 h. The color legend represents the correlation coefficient R value calculated based on the gene expression profiles from microarray study. **B.** Heatmap of GEPs generated by Sparse Hierarchical clustering. The details of the genes in the heatmap are provided in Supplementary Table S3. **C.** The 3 PDXs (1 contaminated and 2 validated) were treated with increasing concentrations of Cisplatin, and viability was assessed by Alamar Blue assay after 48 h incubation. **D.** Time course study of Dexamethasone cytotoxicity on the 3 PDXs (1 contaminated and 2 validated) and viability assessed by 7-AAD exclusion using flow cytometry. Values are expressed as a percentage of the untreated control. Data represent Mean + SEM for N = 3 experiments.

Performing gene-expression microarray studies on the SNP-discrepant PDXs further confirmed the contamination identified by the PAS. The gene expression profiles (GEPs) of two validated ALL-19 PDXs, P5-17 and P5-35, are highly correlated with each other, showing high correlation coefficient R values (red; Figure 5A). The GEP is maintained almost unchanged after 24 h culture *in vitro*. However, the SNP-discrepant ALL-19 PDX, P5-18, shows a very distinct GEP compared to the two validated PDXs (low R value, green). Furthermore, P5-17 and P5-35 are closely clustered together (Figure 5A) and show similar gene expression in the heatmap (Figure 5B), but are mismatched with P5-18.

However, the low level of contamination may be overlooked in studies such as cytotoxicity assays. There was no significant difference between the validated PDXs and the contaminated PDX in their responses to cisplatin and dexamethasone *in vitro* (Figure 5C and 5D). This highlights the importance of validating PDX samples prior to any other studies, as mixed samples are not obvious in many assays.

### Chimeric profiles in samples from patients post-transplant

**Figure 6:**
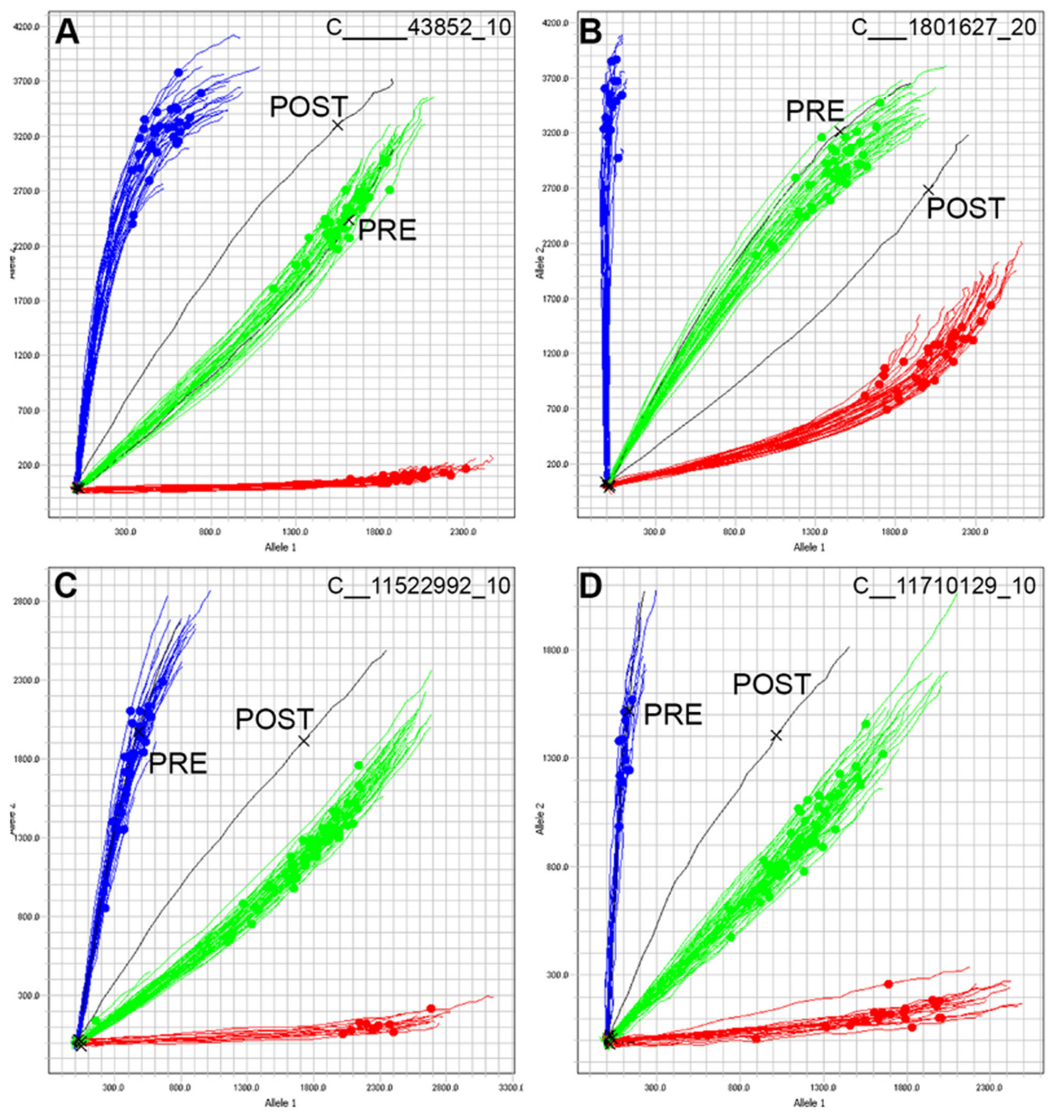
Tracking chimerism in a cancer patient post-transplant DNA from one patient with ALL was SNP genotyped at remission (PRE, black) and seven months following a double cord blood transplant (cord blood derived from two donors, POST, black). **A-D.** Four representative SNPs are shown, and 96 samples were run on this chip. Samples cluster according to their respective genotypes, and the pre-transplant sample PRE clusters as expected. However, the post-transplant sample POST is an outlier in all 4 SNPs, and does not cluster with the remaining samples, confirming the presence of DNA not derived from the original patient.

Bone marrow or cord-blood transplantation is a common treatment option for high-risk leukemia patients, and results in patients with chimeric genotypes in their hematopoietic compartment (patient and donor). Allelic discrimination plots were examined at 4 SNPs for a patient sample before (PRE) and after (POST) a double-cord blood transplant (Figure 6), in which cord blood from two donors was used for transplant. For all SNPs, the pre-transplant sample (PRE) clusters with the other samples on the chip without any signs of imbalance suggesting a pure and single-origin sample. The post-transplant sample (POST) is an outlier, and is shifted away from the PRE sample and all other samples on the chip, suggesting a mixture of DNA in the sample. Thus, as a result of the double-cord transplant, the POST sample for this patient has a chimeric profile that is easily detectable with the PAS platform.

### Complex karyotypes in PDX lines

**Figure 7:**
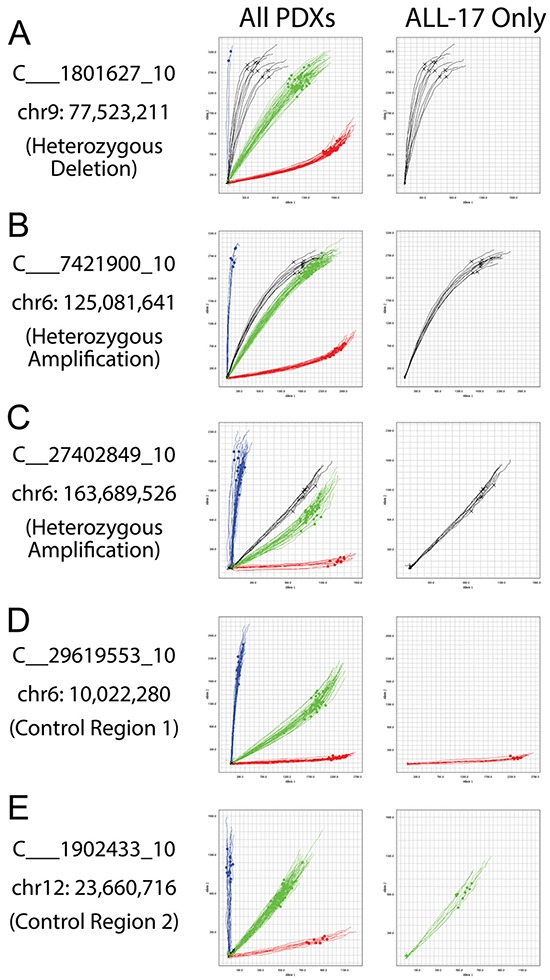
Allelic imbalances detected in patients with complex karyotypes ALL-17 was previously determined to have a heterozygous deletion on chromosome 9 and multiple amplifications on chromosome 6 [28]. **A.** Allelic discrimination plots of a SNP (C___1801627_10) at the heterozygous deletion region on chromosome 9. **B** and **C.** Allelic discrimination plots of two SNPs (C___7421900_10 and C__27402849_10) at the multiple-amplification region on chromosome 6. **D** and **E.** Two SNPs (C__29619553_10 and C___1902433_10) located at the normal genomic regions on chromosome 6 and 12 serve as controls. The left-side plots include a panel of PDXs from several different patients; the right-side plots show ALL-17 only. Black curves, ALL-17 samples; red curves, pure homozygous allele 1 amplifications; blue curves, pure homozygous allele 2 amplifications; green curves, amplifications of heterozygous alleles.

Cancer cells can have complex karyotypes due to chromosomal duplications, deletions and translocations, all of which can impact SNP genotyping. We previously identified a heterozygous deletion of the long arm of chromosome 9 (chr9: 37,380,672 - 113,090,178) and multiple amplifications (copy number = 3) of chromosome 6 (chr6: 30,466,936 - 170,792,391) in the ALL-17 genome (Table 2) [28]. The 32A OpenArray Genotyping chip contains 1 SNP (C___1801627_10) located at the deletion region on the chromosome 9, and 2 SNPs (C___7421900_10 and C__27402849_10) located at the amplification region on chromosome 6 (Table 3). The heterozygous deletion on chromosome 9 in ALL-17 results in abnormal amplification curves of C___1801627_10 located between the pure homozygous allele2 amplifications (blue curves) and the equal amplification of two heterozygous alleles (green curves; Figure 7A). Next, the heterozygous amplification on chromosome 6 results in a shift in amplification of 2 SNPs away from the heterozygous alleles towards allele 2 (Figure 7B and 7C). This indicates that the two SNPs are heterozygous in ALL-17 and there are more copies of allele 2 than allele 1. Moreover, despite shifting away from all other samples on the chip, the amplification curves of these SNPs are consistent across all ALL-17 samples. In contrast, two SNPs located at normal genomic regions on chromosome 6 and 12 serve as controls, showing pure homozygous allele 1 amplification (red curves) and equal amplification of two heterozygous alleles (green curves; Figure 7D and 7E, respectively). Therefore, changes in SNP genotyping profiles due to complex karyotypes should be taken into account during the authentication process.

**Table 2:** Deletion and amplification detected by genome-wide copy number analysis in ALL-17 xenograft samples*

	Chromosomal region		Copy Number
**Heterozygous Deletion**	9p13.2-q31.3	Chr9: 37,380,672 - 113,090,178	1
**Heterozygous Amplification**	6p21.33-q27	Chr6: 30,466,936 - 170,792,391	3

Genomic position refers to NCBI35/hg17.

* Previously published data [28].

**Table 3:** Representative probes detecting genomic regions with copy number variation in ALL-17

	SNP probe	SNP ID	Chromosomal location	Copy number variation
**A**	C___1801627_10	rs10869955	chr9:77,523,211	Heterozygous Deletion
**B**	C___7421900_10	rs1415762	chr6:125,081,641	Heterozygous Amplification
**C**	C__27402849_10	rs6927758	chr6:163,689,526	Heterozygous Amplification
**D**	C__29619553_10	rs9396715	chr6:10,022,280	No
**E**	C___1902433_10	rs10771010	chr12:23,660,716	No

Genomic position refers to NCBI35/hg17.

**Figure 8:**
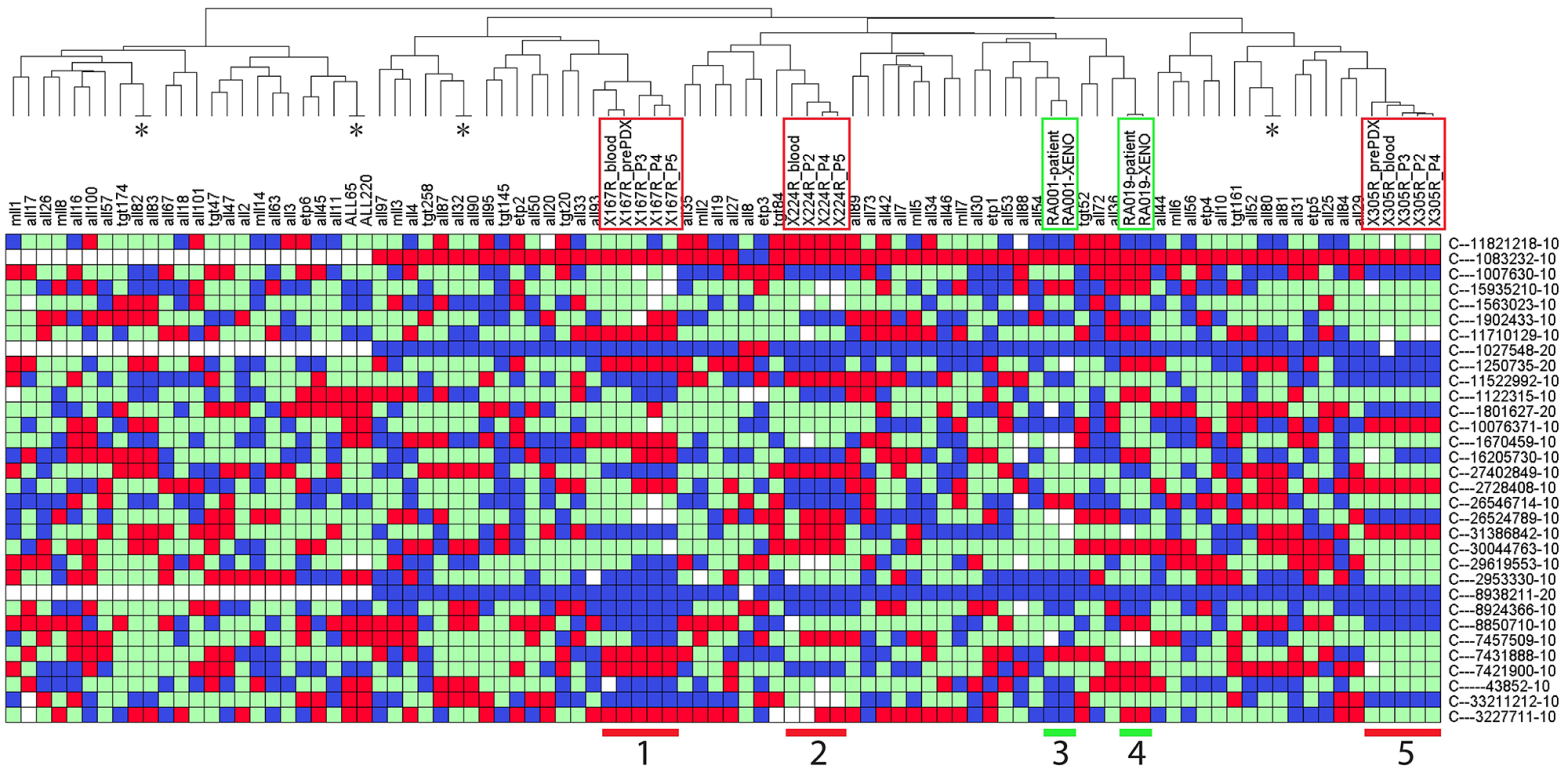
Authentication of solid tumor PDXs using PAS Prostate cancer PDXs from three patients (X167, X224 and X305; groups 1, 2, and 5 with red frames), and Ewing's sarcoma PDXs from two patients (RA001 and RA019; groups 3 and 4 with green frames), were SNP barcoded and clustered with the Reference SNP profiles. See Table 4 for details of the PDXs. In the heatmap, red refers to Homozygous Allele 1, blue refers to Homozygous Allele 2, green refers to Heterozygous, and white refers to no amplification or undetermined PCR. Four pairs of ALL PDXs (labeled with *, ALL-80/-81, ALL-82/-83, ALL-32/-90, ALL-65/-220) match as expected because they were derived from the same patients in independent experiments.

Furthermore, we examined the utility of the PAS to authenticate solid tumor PDX models, such as prostate cancer and pediatric Ewing's sarcoma. Prostate cancer PDXs from three patients and Ewing's sarcoma PDXs from two patients were SNP barcoded and clustered with our reference table (Table 4; Figure 8). In all cases the pre-PDX (including blood and/or original tissues) and matching PDX samples clustered together, which warrants using PAS for authentication of these PDXs. However, for two of the prostate cancer lines (X167R and X224R), there were undetermined SNPs for several probes (4 white squares per PDX on average in Figure 8), and discrepancies in SNP profiles between pre-PDX and matching PDX samples (6 SNPs for X167R and 5 SNPs for X224R). Despite several discrepant SNPs, the SNP profiles were highly consistent in PDX samples derived from the same patient.

**Table 4:** Summary of solid tumor samples

	Xenograft ID	Passage	SNP test ID
**Prostate cancer**	X167R	Blood	X167R_blood
		Pre-PDX*	X167R_prePDX
		Passage 3	X167R_P3
		Passage 4	X167R_P4
		Passage 5	X167R_P5
	X224R	Blood	X224R_blood
		Passage 2	X224R_P2
		Passage 4	X224R_P4
		Passage 5	X224R_P5
	X305R	Blood	X305R_blood
		Pre-PDX	X305R_prePDX
		Passage 2	X305R_P2
		Passage 3	X305R_P3
		Passage 4	X305R_P4
**Ewing's sarcoma**	RA001	Pre-PDX	RA001-patient
		Passage2	RA001-XENO
	RA019	Pre-PDX	RA019-patient
		Passage2	RA019-XENO

* Pre-PDX: primary patient cancer cells that were used to generate PDX of passage 2.

Finally, to assess the reproducibility of the PDX and PAS systems we analyzed 4 pairs of ALL PDXs (ALL-80/-81, ALL-82/-83, ALL-32/-90, ALL-65/-220), each pair being derived from the same patient sample. In each case the PDX SNP analysis was identical (Figure 8).

## DISCUSSION

We have developed a SNP-based PDX authentication system, PAS, that has potential application for the authentication of PDXs. Thirty two SNPs were genotyped using PAS, which generated a patient specific profile that can be used to track patient tissue and identify mixed samples at dilutions as low as 1 in 32. Our observation that SNP profiles were conserved in PDXs established from the same patient in independent experiments (Figure 8) supports the use of SNP analysis for PDX authentication. We successfully identified contaminated PDXs from a panel of ALL-19 samples from various passages engrafted in 74 individual mice. We also identified a patient sample post-transplant with clear evidence of somatic mosaicism due to donor and recipient cells. Furthermore, despite the genomic instability of cancer cells that can cause changes in the SNP genotyping profile, a patient's karyotype can be used to control for these changes. We have generated full genotypes of all commonly used PDXs in our group, and used PAS to validate every PDX sample prior to any other studies.

SNP genotyping for the purpose of human identification is an increasingly accepted technology that offers several advantages over traditional STR profiling. SNPs have a lower mutational rate than STRs, offer higher throughput with rapid automated analysis, are more cost-effective, and offer equivalent discriminatory power [23, 24, 29–31]. The challenge for the implementation of SNP genotyping is the establishment of a standard panel of SNPs that can be used for identification purposes, and that can be readily shared across laboratories. To this end, we chose a commercially available pre-designed panel that can be readily purchased for low or high-throughput applications for the purpose of human identification. The discriminatory power of this panel is such that in a biobank of 10,000 samples, the probability of a random match is less than 0.0005%. As expected, in our biobank of PDXs from 73 patients, all our patient samples were confirmed to be different from each other, and the closest two samples (etp6 and all45) were different in 12 SNPs. Furthermore, the PAS is sufficiently robust to identify mixtures down to 1 in 32, which is similar to the expected ratios of STR profiling [17, 22]. These data demonstrate that the detection of contaminated PDXs by PAS is possible, however, compared to STR, the identification of the contaminating PDX by PAS is challenging.

SNP genotyping allows for more extensive validation of a biobank compared to STR profiling. Based on cost estimates and time commitment, our PAS system costs approximately US$7.20 per sample, while STR usually costs around US$20–30 per sample. In addition, sample handling for 96-samples is 50 min for PAS and 3 h for STR, and machine run times for 96-samples are 3.5 h for PAS versus 18 h for STR [17, 32]. In practice, this means that SNP validation is sufficiently cost effective to validate every sample in an experiment, rather than using STR validation of one representative sample per experiment. While the cause of a mis-identified sample is often difficult to determine, our experience with SNP validation has shown that it is important to validate all the samples from an experiment to prevent one mis-identified sample from contaminating the entire PDX line.

SNP genotyping offers authentication in cancer-derived xenografts that have high genomic instability. Genetic drift is commonly reported in cell lines that have been extensively cultured or that exhibit deficiencies in mismatch repair, resulting in changes in the karyotype or the gene loci that can affect the STR profile [17, 22]. Similarly, cancer cells can have complex karyotypes that can confound the results of STR analysis. While SNP genotyping is not immune from such changes in the genetic profile, SNPs have a much lower overall mutational rate than STRs (2.5 × 10^-8^ vs 10^-3^ to 10^-4^), reducing the chances of spontaneous change [33]. Furthermore, changes in a SNP profile are more likely to be the result of whole or partial chromosome changes that can be detected in a patient's karyotype. One example is using PAS to authenticate PDXs with complex karyotypes, such as ALL-17 and several solid tumors. Several undetermined SNPs (shown as white squares) and discrepancies between pre-PDX and PDX samples were identified, which could be due to 1) complex karyotypes of these PDXs as discussed in Figure 7, or 2) genomic differences between the normal and cancer cells and the infiltration with normal tissues in solid tumor samples. Nevertheless, SNP profiles across passages of PDXs were consistent, suggesting that white squares in PDXs can be interpreted as correct barcodes, as long as they are consistent from primary xenografts to high passage xenografts.

Cell line authentication has become a routine procedure in most laboratories to track mis-identified cell lines, and is a requirement for publication in many journals [17]. However, journals currently do not enforce a similar standard on PDXs, which can have the same issues of mis-identification and contamination as cell lines. PDXs are frequently used in animal experiments that are more resource intensive compared to experiments involving cell lines, and the data generated following animal xenografting experiments are increasingly being linked to patient clinical outcome [1, 8] and can be used to direct treatment [34]. In our study, although the majority of SNPs were consistent, 10% were identified as discrepant in ALL-19 PDXs (8 passage-5 of 74 PDXs) using the PAS platform, indicating the need to monitor this at high numbers of passage. These contaminated PDXs showed similar responses to validated PDXs in cytotoxicity assays. While this type of contamination may be rare with solid tumor PDXs since they are often propagated as tumor pieces, the PAS may still be helpful for eliminating human error during labeling and archiving. Therefore, the authentication of PDXs is a particularly important quality control process. The advent of PAS has made this process highly cost-effective and rapid, enabling more routine screening of PDXs.

## MATERIALS AND METHODS

### Ethical statement and ALL patient tissue

ALL specimens were obtained for xenografting with informed, written consent approved by the Human Research Ethics Committees of the South Eastern Sydney Illawarra Area Health Service (HREC10114) and the University of New South Wales (HREC10442), and the Animal Care and Ethics Committee of the University of New South Wales (15/105B). The release of both primary ALL cells for xenografting and primary patient DNA samples for this study was approved by the Children's Cancer Institute Australia Tumor Bank Management Committee.

### Preparation of patient ALL samples and isolation of DNA

Bone marrow aspirates (2-4 mL) were collected in Acid Citrate Dextrose tubes (Becton Dickinson, USA) from anaesthetized pediatric patients for the primary purpose of leukemia diagnosis and minimal residual disease (MRD) diagnostics. Immediately after collection in local hospitals, the bone marrow aspirates were transported at room temperature to the laboratory and mononuclear cells purified by density gradient centrifugation. The surplus mononuclear cells were aliquoted and cryopreserved in fetal calf serum with 10% DMSO in liquid nitrogen.

### Establishment of ALL PDXs

PDXs were established based on a previously published protocol [2, 7, 8]. Briefly, patient mononuclear cells purified as described above were inoculated into nonobese diabetic/severe combined immunodeficient NOD/SCID or NOD/SCID/IL-2 receptor gamma negative (NSG) mice by tail vein injection. Engraftment was monitored weekly by flow cytometry analysis of human CD45 cells in the peripheral blood. Mice were euthanized if they showed overt signs of leukemia. Mononuclear cells were harvested from the spleen as described above.

### Establishment of PDXs from solid tumors

Full details of ethical statements and methods of establishing PDXs from prostate cancer [35] and Ewing's sarcoma patient samples are provided in the Supplemental Methods.

### DNA extraction

Genomic DNA was extracted from cell pellets of approximately 5 million cells using the PureLink Pro 96 Genomic DNA Kit (K1821-04A, Life Technologies, USA), as per the manufacturer's instructions. DNA was quantified with the QuantiFluor dsDNA dye (E2670, Promega, USA).

### SNP genotyping

Purified genomic DNA (150 ng) was amplified using the TaqMan 32A OpenArray Genotyping Barcode Panel (4475386, Life Technologies, USA). Samples were loaded using the AccuFill system, and amplification performed on the QuantStudio 12K Flex Real-Time qPCR instrument. The QuantStudio 12K Flex software was used to manually determine genotype profiles for 32 chosen SNPs, and the data exported for further analysis. The QuantStudio 12K Flex performs a multicomponent analysis based on the qPCR curves for each bi-allelic assay, and generates a real-time allelic discrimination plot. Homozygous alleles amplify on the *x* or *y* axis, with heterozygous alleles amplifying along the *x* = *y* slope.

### Data analysis using in-house R studio programs

SNP genotype profiles of the PDX samples were exported into an Excel file and analyzed using in-house R scripts written with R Studio. Briefly, a reference table of patient or the earliest available PDX passage genotype profiles was generated using a script “Assign References.r”. Any new reference profile could be added to the existing reference table by a script “AddCalls.r”. Matching the PDXs to their reference SNP genotype profile was done using a script “Validation function.r”. This would generate 3 text files, including a summary of genotyping results, a list of contaminated samples, and a list of validated samples. Next, a heatmap could be generated using a script “OneFunction heatmap.r”. All R scripts are provided in the Supplementary Materials.

### Microarray analysis

For gene expression and microarray studies, RNA was extracted using the RNeasy Mini Kit (Qiagen, USA). The microarray analysis of gene expression was performed using Illumina HumanHT-12 chips and analyzed using GenePattern as previously described [36–41]. The data were deposited in NCBI's Gene Expression Omnibus [42] (GSE74299).

### Assessment of chemodrug sensitivity

PDXs were cultured in QBSF 60 medium (Quality Biological, USA) supplemented with Flt-3 ligand (20 ng/mL, ProSpec, USA), penicillin (100 U/mL), streptomycin (100 μg/mL) and L-glutamine (2 mM) (QBSF 60/F). Cisplatin and dexamethasone sensitivities were assessed by measuring mitochondrial activity via the Alamar Blue assay or cell viability by 7-AAD exclusion using flow cytometry as previously described [10, 12, 40, 41]. Cell viability was expressed as a percentage of vehicle treated controls.

## SUPPLEMENTARY TABLES






